# Supplementary material for: Challenges in antenatal care utilization in Kandahar, Afghanistan: A cross-sectional analytical study
Source: PLoS One. 2022 Nov 21;17(11):e0277075. doi: 10.1371/journal.pone.0277075 (PMC9678260; doi:10.1371/journal.pone.0277075)
Supplement: S1 File — (DOCX) [file pone.0277075.s001.docx]

**Questionnaire**

|  |  |  |  |  |  |  |
| --- | --- | --- | --- | --- | --- | --- |

Participant code:

|  | 1. First part: General information |
| --- | --- |
|  | - 1. Name: |
|  | - 1. Age: |
| Primary = 1  Secondary=2  Religious = 3 | - 1. Education: |
| Employed= 1  Non-Employed= 2  Other= 3 | - 1. Job: |
|  | - 1. Monthly income (AF per month) |
| Urban= 1  Rural= 2 | - 1. Where do you live? |
|  | - 1. Address |
| 2. Second part: Services related information | |
| Yes= 1  No= 2 | - 1. Is there any near clinic to your home? |
| Public= 1  Private=2 | - 1. Which one is the nearest clinic to you? |
| Yes= 1  No =2  (If the answer is yes, don’t ask question 4) | - 1. Do you get services from the nearest clinic? |
| it’s far= 1  There is no medicine= 2  There is no night duty= 3  Dr. or MW has no good behavior= 4  My family doesn’t let me= 5  No obeying Moharamyat= 6 | - 1. Why don’t you utilize the services from the nearest health facility? |
| Half or less than half hour= 1  From half to one hour= 2  From one to two hours= 3  More than= 4 | - 1. How much is the distance from your home to clinic? |
| Mother and child health care=1  children health care= 2  Vaccination= 3  Infectious disease, such as, TB and Malaria=4  Mental disease, like, depression, anxiety= 5 | - 1. What do you think which kind of services do the clinics provide? |
| ANC= 1  Delivery= 2  PNC= 3  For vaccination= 4  To get family planning methods = 5  For TB treatment= 6  other disease treatment= 7 | - 1. For what purpose you have visited the clinic for the last time ? |
| Yes= 1  No= 2 | - 1. Have you gotten the needed services? |
| Very Good= 1  Good = 2  Not good= 3  I don’t know/ I have no Idea= 4 | - 1. How was the behavior of clinic/hospital personnel? |
| One year or smaller= 1  Two years= 2  Bigger than 2 years= 3 | - 1. How old is your last child? |
| Once= 1  Twice= 2  Three time= 3  Four time=4  I don’t know= 5 | - 1. How many times your last child been vaccinated? |
| Oral Drops= 1  Injection in left arm after birth = 2  Injection in leg= 3  I don’t know= 4 | - 1. Which vaccine has your child gotten? |
| tube wells= 1  Piped water =2  Springs= 3  Rainwater= 4 | - 1. What is the main source of drinking water for members of your household? |
| Yes=1  No= 2 | - 1. Do you do anything to become water safer? |
| Boil= 1  Add bleach/chlorine= 2  Strain through a cloth use water filter (ceramic/sand/composite/etc.)= 3  water filter = 4  solar = 5  other =6 | - 1. What do you usually do to make the water safer to drink? |
| 3. Third part: If your interviewee was female, you may ask them below questions. | |
| 1 = 1  (2-5)= 2  >5 = 3 | - 1. How many times you have delivered? |
| Yes = 1  No= 2 | - 1. Do you have visited female doctor of Midwife for ANC visit during pregnancy? |
| Once = 1  (2-4) times =2  4> times= 3 | - 1. How many times you have visited HFs for ANC visit during your last pregnancy. |
| 1= 1  (2-5) = 2  5> = 3 | - 1. How many alive children do you have? |
| Home= 1  Public clinic= 2  Private clinic= 3  (if the answer was 1, ask question, if the answer was 3 don’t ask question 7) | - 1. Where have you had your last delivery? |
| Hospital/public/private clinic ask for money and we have no money= 1  Hospital/public/private clinics  are far away= 2  Hospital/public/private clinic personnel have no good Behavior = 3  My family doesn’t let me= 4 | - 1. Why have you had your delivery in home? |
| Public clinic has no good delivery room= 1  Public clinic/hospital personnel have no good Behavior=2  Moharamyat isn’t considered in public clinic/hospital= 3  There is no body in public clinic/hospital at night shift= 4  Public clinic/hospital is far away= 5  Public clinic/hospital ask for money= 6 | - 1. Why have you had your delivery in private clinic/ hospital? |
| Within one hours= 1  After (1-2) hours = 2  After(2- 5) hours = 3 | - 1. When you start your Brest feeding to your newborn baby? |
| Less than 6 months= 1  Up to 6 months = 2  More than 6 months = 3 | - 1. How long you are feed your baby only on your Brest milk? |
| Very good= 1  Good= 2  Fair= 3  Bad= 4  Very bad= 5 | - 1. How was the Behavior of midwife and clinic personnel? |
| Yes = 1  No= 2 | - 1. Do you used family planning methods? |
| Condom= 1  POP = 2  CoC= 3  IUD= 4  LAM = 5  Injection= 6 | - 1. Which method do you used? |
| Condom= 1  POP = 2  CoC= 3  IUD= 4  LAM = 5  Injection= 6 | - 1. Which family planning method is satisfactory for you? |
|  | - 1. What do you think what the greatest trouble in a public clinic/hospital is? |
