# Supplementary material for: Challenges in antenatal care utilization in Kandahar, Afghanistan: A cross-sectional analytical study
Source: PLoS One. 2022 Nov 21;17(11):e0277075. doi: 10.1371/journal.pone.0277075 (PMC9678260; doi:10.1371/journal.pone.0277075)
Supplement: Attachment — Submitted filename: Review.pdf [file pone.0277075.s003.pdf]

I thank the editors for the opportunity to review the paper.

- The authors presented an original study from Afghanistan where they used primary data collection to assess factors affecting maternal health care utilisation in one city.
- Primary data is collected using a cross-sectional survey design from 1500 women approximately who are attending antenatal care clinics and provided an opportunity to respond to an interview where questions of social demographic and health system factors were inquired. Quantitative techniques using univariate and bivariate regression were used to assess the association of factors and likelihood of using anti-Natal care, which was the main outcome. corroborating global evidence, the authors find that ante Natal care use in the study area is linked to factors such as behaviour and attitude of health workers social-economic status of the mother. Afghanistan is one of the global high burden countries where maternal mortality is still substantive presents a unique opportunity to understand factors affecting maternal health in the country. The authors, by conducting the study, try to fill in the knowledge gap that policymakers we like to review to improve the quality of health services.

- Overall while the paper has the potential to be published in its current form, it has key issues in the framing of the research question, the presentation of the results the discussion that affects publication. I am sharing below key comments for the authors to consider to improve the manuscript

Background:

- The global maternal health status has improved over the last two decades with substantive improvements during the UN global goals namely the MDG and over the SDG. In the background section, the authors may like to consider that Afghanistan's challenges have been unique in consideration of ongoing conflicts which have severely affected not only the capacity of the health services to deliver quality maternal health care but also the broader disruption to the social determinants of health. I would encourage the authors to review the background and rephrase the background 2 compare Afghanistan's progress with similar high burden high conflict countries and provide details of specific programmes that the country may have initiated that are expected to improve maternal health services.

Methods:

- The authors may also like to provide a better rationale for the selection of the region Kandahar and provide sampling details as to how women were chosen from the specific antenatal clinics to participate in the study. women choosing to attend antenatal care services Its likely to be different from those that do not attend services in the first place.
- In the method section, I would also like to see a description of the outcome and explanatory variables as well as confounding variables. in the resulting discussion for

example in Table 3, the authors mentioned health clinic staff behaviour. However, it was not explained what this question actually comprises. play authors does may like to dedicate sections under methods to talk about the key explanatory variables and they can structure it using the social determinants of health or the social-ecological framework where individual household community and health system factors are separately described.

Discussion and Conclusion

- The authors may like to provide a limitation section in the discussion section.
- Additionally, the current presentation of studies in the discussion section in Table 5 is highly disruptive and not suited photo paper of this nature. please review key information from Table 5 that you may like to present in the narrative form and add on in the discussion section.
- A common comment for the paper formatting especially the tables is that the authors may like to review the journal's formatting requirement and reduce the number of tables as well as adhere to the journal's formatting requirement
