# Supplementary material for: Challenges in antenatal care utilization in Kandahar, Afghanistan: A cross-sectional analytical study
Source: PLoS One. 2022 Nov 21;17(11):e0277075. doi: 10.1371/journal.pone.0277075 (PMC9678260; doi:10.1371/journal.pone.0277075)
Supplement: Attachment — Submitted filename: Response to the Reviewers.docx [file pone.0277075.s004.docx]

PONE-D-21-18840R1

Title of the Article: Challenges in antenatal care utilization in Kandahar, Afghanistan: A cross-sectional analytical study.

Bilal Ahmad Rahimi

Dear Dr. Rahimi,

We've checked your submission and before we can proceed, we need you to address the following issues:

1. Please upload a Response to Reviewers letter which should include a point by point response to each of the points made by the Editor and / or Reviewers. (This should be uploaded as a 'Response to Reviewers' file type.) Please follow this link for more information: [http://blogs.PLOS.org/everyone/2011/05/10/how-to-submit-your-revised-manuscript/](http://blogs.plos.org/everyone/2011/05/10/how-to-submit-your-revised-manuscript/)

Answer: OK. A “Response to Reviewers” file has been uploaded.

2. Please include additional information regarding the survey or questionnaire used in the study and ensure that you have provided sufficient details that others could replicate the analyses. For instance, if you developed the survey or questionnaire as part of this study and it is not under a copyright more restrictive than CC-BY, please include a copy, in both the original language and English, as Supporting Information. If the questionnaire is published, please provide a citation to the (1) questionnaire and/or (2) original publication associated with the questionnaire.

Answer: OK. Now questionnaire has been uploaded as Supporting Information.

3. Please revise the title of your manuscript to remove "Title of the Article:" in the submission system.

Answer: Now "Title of the Article:" is removed in the submission system.

4. Thank you for stating the following financial disclosure: "There was no financial support for this research from any source."

At this time, please address the following queries:

a) Please clarify the sources of funding (financial or material support) for your study. List the grants or organizations that supported your study, including funding received from your institution.

b) State what role the funders took in the study. If the funders had no role in your study, please state: “The funders had no role in study design, data collection and analysis, decision to publish, or preparation of the manuscript.”

c) If any authors received a salary from any of your funders, please state which authors and which funders.

d) If you did not receive any funding for this study, please state: “The authors received no specific funding for this work.”

Please include your amended statements within your cover letter; we will change the online submission form on your behalf.

Answer: We did not have any funding sources for our study. The publication fee for our article will be provided by MORU (Mahidol-Oxford Tropical Medicine Research Unit), Bangkok. MORU will pay only if the journal put the following statement in the funding part of the article:

“This study did not receive any specific funding. WR Taylor is part funded by Wellcome under grant 220211. For the purpose of Open Access, the author has applied a CC BY public copyright licence to any Author Accepted Manuscript version arising from this submission.”

I have mentioned this in the Cover letter too.

5. Thank you for stating the following in your Competing Interests section:  "There are no competing interests."

Please complete your Competing Interests on the online submission form to state any Competing Interests. If you have no competing interests, please state "The authors have declared that no competing interests exist.", as detailed online in our guide for authors at <http://journals.plos.org/plosone/s/submit-now>

This information should be included in your cover letter; we will change the online submission form on your behalf.

Answer: We have added the following sentence in the Cover letter:

“The authors have declared that no competing interests exist.”.

6. In your Data Availability statement, you have not specified where the minimal data set underlying the results described in your manuscript can be found. PLOS defines a study's minimal data set as the underlying data used to reach the conclusions drawn in the manuscript and any additional data required to replicate the reported study findings in their entirety. All PLOS journals require that the minimal data set be made fully available. For more information about our data policy, please see http://journals.plos.org/plosone/s/data-availability.

Upon re-submitting your revised manuscript, please upload your study’s minimal underlying data set as either Supporting Information files or to a stable, public repository and include the relevant URLs, DOIs, or accession numbers within your revised cover letter. For a list of acceptable repositories, please see <http://journals.plos.org/plosone/s/data-availability#loc-recommended-repositories.> Any potentially identifying patient information must be fully anonymized.

Important: If there are ethical or legal restrictions to sharing your data publicly, please explain these restrictions in detail. Please see our guidelines for more information on what we consider unacceptable restrictions to publicly sharing data: <http://journals.plos.org/plosone/s/data-availability#loc-unacceptable-data-access-restrictions.> Note that it is not acceptable for the authors to be the sole named individuals responsible for ensuring data access.

We will update your Data Availability statement to reflect the information you provide in your cover letter.

Answer: As the data file is the property of Kandahar University Research Center, we are not allowed to share the data file with anyone. For this we contacted the research center. They provided a part of the data from the main SPSS data file. Now have uploaded it as the Supporting Information file.

Also, we mentioned this information in the Cover letter.

Thank you for submitting your work to PLOS ONE and supporting our mission of Open Science.

Kind regards,

Richard Ibañez Dilla

PLOS ONE

Many thanks

Bilal
