# Supplementary material for: Challenges in antenatal care utilization in Kandahar, Afghanistan: A cross-sectional analytical study
Source: PLoS One. 2022 Nov 21;17(11):e0277075. doi: 10.1371/journal.pone.0277075 (PMC9678260; doi:10.1371/journal.pone.0277075)
Supplement: Attachment — Submitted filename: Response to the Reviewers.docx [file pone.0277075.s005.docx]

PONE-D-21-18840R1

Title of the Article: Challenges in antenatal care utilization in Kandahar, Afghanistan: A cross-sectional analytical study.

Bilal Ahmad Rahimi

- Please also amend your Methods section to include details of how the questionnaire was developed, tested and validated.

Answer: Now details have been added in the “Materials and methods” section (lines 181-189) to clarify how the questionnaire was developed, tested and validated.

Review Comments to the Author

Please use the space provided to explain your answers to the questions above. You may also include additional comments for the author, including concerns about dual publication, research ethics, or publication ethics. (Please upload your review as an attachment if it exceeds 20,000 characters)

Reviewer #1: Dear Authors,
Thank you for submitting an improved version.

The response to my previous comments has been attempted but has not been satisfactorily completed.

1. The background section for example starts with a summary of MDG's and the SDG goal' which is not needed and does not add value to the paper. Rather, Afghanistan's MMR progress in the SDG and MDG is important to highlight.

Answer: Summary of MDGs and SDGs goals have been removed from the “Introduction” section. Afghanistan's MMR progress in the SDG and MDG is now added in the second last paragraph of the “Introduction”.

2. Similarly, the study variables on page 8 lines 193 need better explanation of why they were chosen with links to the literature.

Answer: Now the study variables on page 8 have explained why they were chosen and also liks to the literatures have been provided.

3. The definitions sections in page 9 also needs work-Please provide narrative explanation of these definitions along with links to the literature.

Answer: Now the definitions section has been explained narratively and links are provided.

4. Formatting of table 3 remains an issue. I would encourage the authors to make these changes in depth as the paper provides important findings.

Answer: I formatted table 3. To make table 3 clearer and easy to understand, I merged tables 3 and 4 as one table. Now this table contain both Chi-square analyses and logistic regression, making it very easy to understand.

Many thanks

Bilal
