# Supplementary material for: Challenges in antenatal care utilization in Kandahar, Afghanistan: A cross-sectional analytical study
Source: PLoS One. 2022 Nov 21;17(11):e0277075. doi: 10.1371/journal.pone.0277075 (PMC9678260; doi:10.1371/journal.pone.0277075)
Supplement: Attachment — Submitted filename: Response to the Reviewers.docx [file pone.0277075.s006.docx]

PONE-D-21-18840R1

Title of the Article: Challenges in antenatal care utilization in Kandahar, Afghanistan: A cross-sectional analytical study.

Bilal Ahmad Rahimi

Review Comments to the Author

Dear Authors,

Thanks for submitting your research with PLOS One. While the paper provides important insights to challenges of ANC service utilization in Kandahar, Afghanistan; there are certain key issues which must be addressed beforehand for possible publication.

Introductions:

• The first paragraph could be shortened and should be written more focussed on Afghanistan; the irrelevant information can be removed.

- Answer: OK. Now the first paragraph has been shortened, irrelevant information has been removed, and now focussed on Afghanistan.

• 92 Generalized sentences like- ‘International community has always tried to reduce maternal mortality’ should be converted to more specific ones.

- Answer: OK. Now these generalized sentences have been removed and converted into more specific sentences.

Methodology:

• 152.Study Design should be mentioned more specifically (almost all quantitative studies are questionnaire based)

- Answer: OK. Thanks for the comment. To make it clearer and specific, now I changed it to “Cross-sectional questionnaire-based study” .

• 156. Why these four clinics were chosen in Kandahar city should be explained properly.

- Answer: OK. Now it is clearly explained. “These health clinics were randomly selected using lottery-method.”

• 160. Why Kandhar province was selected for the study should move up in the study methodology, probably to line No. 156.

- Answer: OK. As per reviewer comment, now this sentence is moved up to line 156.

• 170.Why unmarried female were excluded and how ethical consideration were met for them may be discussed briefly?

- Answer: Now the following sentences have been added in exclusion criteria after “unmarried females”:

“These females are excluded due to the facts that extramarital pregnancies are rare and also considered very big sin in the Afghan society. If the family members get information of extramarital pregnancy, there is a fear that the female can be tortured or even killed.”

• 203. The Independent variable section n methodology does not mention Breastfeeding time of last child or any other variable related to ‘Using methods to make drinking water safe’; but they have been included in the result tables 2 &3. Also, how these variables can affect ANC services utilization can be briefly addressed for wide comprehension. If deemed not suitable these variables should be removed from the result tables.

- Answer: OK. Thanks for the good point. As these 2 variables (i.e., “Breastfeeding time of last child” and “Using methods to make drinking water safe” do not affect ANC services utilization, now they have been removed from the tables.

Formatting of intext citation should be checked and corrected as per journal policy.

- Answer: OK. Thanks. Now the intext citation is thoroughly checked and corrected as per journal policy. For example, intext citation of 1,2,3,4 has been changed to 1-4.

Many thanks

Bilal
